# Altered Hematopoiesis in Mice Lacking DNA Polymerase μ Is Due to Inefficient Double-Strand Break Repair

**DOI:** 10.1371/journal.pgen.1000389

**Published:** 2009-02-20

**Authors:** Daniel Lucas, Beatriz Escudero, José Manuel Ligos, Jose Carlos Segovia, Juan Camilo Estrada, Gloria Terrados, Luis Blanco, Enrique Samper, Antonio Bernad

**Affiliations:** 1 Departamento de Inmunología y Oncología, Centro Nacional de Biotecnología, Consejo Superior de Investigaciones Cientificas, Campus Universidad Autónoma de Madrid, Cantoblanco, Madrid, Spain; 2 Departamento de Cardiología Regenerativa, Unidad de Celómica, Centro Nacional de Investigaciones Cardiovasculares Carlos III, Madrid, Spain; 3 Hematopoiesis and Gene Therapy Division, Centro de Investigaciones Energéticas, Medioambientales, y Tecnológicas, Centro de Investigación Biomédica en Red de Enfermedades Raras, Madrid, Spain; 4 Centro de Biología Molecular Severo Ochoa, Consejo Superior de Investigaciones Cientificas, Campus Universidad Autónoma de Madrid, Cantoblanco, Madrid, Spain; University of North Carolina at Chapel Hill, United States of America

## Abstract

Polymerase mu (Polμ) is an error-prone, DNA-directed DNA polymerase that participates in non-homologous end-joining (NHEJ) repair. *In vivo*, Polμ deficiency results in impaired Vκ-Jκ recombination and altered somatic hypermutation and centroblast development. In Polμ^−/−^ mice, hematopoietic development was defective in several peripheral and bone marrow (BM) cell populations, with about a 40% decrease in BM cell number that affected several hematopoietic lineages. Hematopoietic progenitors were reduced both in number and in expansion potential. The observed phenotype correlates with a reduced efficiency in DNA double-strand break (DSB) repair in hematopoietic tissue. Whole-body γ-irradiation revealed that Polμ also plays a role in DSB repair in non-hematopoietic tissues. Our results show that Polμ function is required for physiological hematopoietic development with an important role in maintaining early progenitor cell homeostasis and genetic stability in hematopoietic and non-hematopoietic tissues.

## Author Summary

Double-strand breaks (DSB) in DNA are a highly deleterious type of genetic damage, potentially causing genomic rearrangements or cell death if unrepaired. DSB can be triggered by environmental factors (such as electromagnetic radiation or clastogenic chemicals) or normal cell metabolism. The main mechanism of DSB repair in mammals is thought to be the non-homologous end-joining (NHEJ) pathway. Our article describes how DNA polymerase mu (Polμ), a recently identified component of the NHEJ machinery, is required for hematopoiesis—the process that generates and maintains the correct balance of the millions of blood cells needed to sustain life and defend against infection. Hematopoietic stem cells (HSC) divide asymmetrically, yielding another HSC and a progenitor cell. These progenitors proliferate and differentiate, their progeny eventually generating mature blood cells. In mice in which Polμ is genetically eliminated, we found that hematopoietic progenitors proliferate slowly and are functionally impaired. The incidence of DSB in hematopoietic cells from these mice is increased, suggesting that reduced DNA repair may be the cause of the hematopoietic defects. DNA damage was also increased in tissues unrelated to hematopoiesis, including liver, kidney, lung, and mouse embryonic fibroblasts. Thus, these results demonstrate that Polμ plays an important role in general DSB repair in many cell lineages.

## Introduction

In higher eukaryotes, DNA double strand breaks (DSB) are repaired through two main pathways: homologous recombination [1] and non-homologous end-joining (NHEJ) (reviewed [2]–[4]). Although NHEJ predominates during the G0/G1 and early S phases of the cell cycle [5],[6], both mechanisms can act in coordination [7]–[9].

Polymerase mu (Polμ) is an error-prone DNA-directed DNA polymerase belonging to the PolX family, with a high amino acid similarity to TdT [10]–[13] and, like TdT, Polμ has terminal transferase activity [11]. Unlike TdT, Polμ is expressed in many tissues (liver, kidney, lung, brain, testis), although it is especially abundant in lymphohematopoietic organs [11],[14]. Polμ interacts with components of the NHEJ repair pathway (Ku 70/80) and is up-regulated after the induction of DSB by γ-irradiation, a known clastogen [15]. Double immunostaining for Polμ and phosphorylated γ-H2AX, a modified histone found at DSB sites, shows that Polμ is recruited to these sites [15]. *In vitro*, Polμ participates in specific NHEJ reactions that require DNA polymerase activity [15]–[17].

Analysis of a Polμ knockout mouse model has identified a specialized function for this enzyme during V(D)J recombination [18]. Specifically, Polμ is required for correct recombination of the immunoglobulin κ light chain during B cell development, and its deficiency results in shorter, non-productive Vκ-Jκ junctions and hence lymphocyte cell death at the transition from PreB to Immature B cell stage [18]. Because V(D)J recombination is essentially a modified end-joining reaction (reviewed in [19]), the participation of Polμ in this process demonstrates its requirement for at least a subset of NHEJ reactions *in vivo*. This role of Polμ has also been demonstrated *in vitro* [16].

The expression pattern of Polμ [11],[14] suggests that it might participate in NHEJ in other tissues, especially in non-B cell hematopoietic populations. In recent years it has been demonstrated that several DNA repair pathways play a clear role in the maintenance of the hematopoietic system and of hematopoietic stem cell (HSC) number and function during aging [20],[21]. For example, in Ercc1-deficient mice hematopoiesis is impaired and bone marrow cellularity and hematopoietic progenitor cell (HPC) numbers are reduced [22]; in mice deficient in Brca2, the reconstitution capacity of HSC and progenitor cells is reduced [23]. Hypomorphic Rad50 mutant mice have growth defects and are predisposed to cancer and progressive HSC failure [24]. Irradiated Parp^−/−^ mice are myelosuppressed, display extensive hemorrhaging and show altered extramedullary hematopoiesis during the recovery phase [25]. Mice deficient either in XPD (from the nucleotide excision repair pathway) or Ku80 (NHEJ) show reduced HPC numbers in bone marrow. Although the numbers of HSC in these mice is unaltered, HSC reconstitution potential during aging is reduced [21]. A similar phenotype is observed in another mouse model of NHEJ deficiency (Lig4^Y288C^ mice; [20]), and together these reports strongly suggest that HSC accumulate mutations during aging that impair their function and highlight the role of the different repair pathways in maintaining hematopoietic homeostasis [20],[21].

We have investigated the role of Polμ in hematopoiesis and general DNA repair. Polμ^−/−^ mice have reduced numbers of most peripheral blood populations and show impaired hematopoiesis as a result of the reduced numbers of hematopoietic progenitors. Polμ^−/−^ hematopoietic and non-hematopoietic cells accumulate DSB, both spontaneously and after γ-irradiation, indicating a role for Polμ in general DSB repair.

## Materials and Methods

### Mice

The generation of Polμ^−/−^ mice has been described previously [14]. Mutant and wildtype (wt) mice were bred in our specific pathogen-free facilities and were routinely screened for pathogens. Most experiments were carried out with animals in the mixed original (129/Balb/c) background. Where indicated, experiments were carried out in the C57BL/6 background. B6.SJL mice, were purchased from The Jackson Laboratory (Bar Harbor, Maine), and housed at the CIEMAT animal facility. All experiments were performed according to Spanish and European regulations for the use and treatment of experimental animals, with the approval of the Centro Nacional de Biotecnología (CNB) and the Fundación Centro Nacional de Investigaciones Cardiovasculares (CNIC) animal ethics committees.

### Bone Marrow and Spleen Cell Suspensions

Femurs and tibias were removed from mice, and bone marrow was extracted by complete flushing with PBS under sterile conditions. Spleens were retrieved and disaggregated in sterile PBS. In each case, tissue extracts were incubated in 0.85% NH_4_Cl to lyse erythrocytes. Cells were counted with a hemocytometer and used in subsequent assays.

### Hematological, Histological, Flow Cytometry, and Cell Cycle Analyses

Blood was collected in a capillary tube from the retro-orbital sinus and transferred to EDTA-coated tubes. For hematology, 25 µl were analyzed in an MS9 machine (Kemia, Madrid, Spain). For flow cytometry (FCM), whole blood was incubated with the corresponding antibodies, fixed, and erythrocytes lysed with OptiLyse C (Immunotech). Cells were analyzed in a Cytomics FC 500 cytometer (Beckman Coulter). Bone marrow cells were stained with antibodies in ice-cold PBS, 0.5% BSA, 2 mM EDTA. Antibodies used were anti-CD19-PE, -CD19-SPRD, -CD3-FITC, -CD4-SPRD, -CD8-PE, -CD11b (Mac1)-PE, -Gr1-FITC, -B220-FITC, -B220-PE, -IgM-PE, -Ter119-PE, -CD41-FITC, (BD-Pharmingen). Cell cycle status was monitored by estimating total cell DNA content in a flow cytometer after staining 70% methanol fixed cells with 10 µg/ml propidium iodide and 10 µg/ml RNase A.

For bone marrow progenitor analysis, bone marrow cells were extracted from femurs and tibias, counted, and stained in ice-cold PBS, 0.5% BSA plus 2 mM EDTA. Except for samples for detection of CMPs, GMPs and MEPs, samples were blocked with FcBlock (anti-CD16/CD32, BD Pharmingen). Samples were stained with the following antibodies: biotin-conjugated lineage antibody cocktail (anti-CD3e, -CD11b, -B220, -Ly6G/Ly6C and -TER-119), anti-CD117 (c-kit) (FITC or PE), anti-CD16/32-FITC, anti-CD135-PE, anti-Sca1-PE-Cy7 (all from BD Pharmingen); anti-CD127-Biotin, anti-CD127-PE (eBiosciences); anti-CD34-PE-Cy5 (Biolegend), and Streptavidin-Pacific Blue (Invitrogen). To exclude dead cells Hoechst 33582 was added to every sample. All samples were acquired with a BDFASCanto II cytometer (BDBiosciences). At least 1 million cells were acquired for each sample. Data were analyzed with FlowJo software (Tree Star, Inc.) and fluorescent-minus-one controls were used to define gates [26].

For histology, femurs or tibias were formalin-fixed, decalcified and paraffin-embedded. Sections (5 µm) were hematoxylin-eosin stained. Photographs were acquired with a Leica DM RB microscope fitted with a DP 70 digital camera (Olympus) and linked to DP controller software (Olympus).

### Splenocyte Culture and Irradiation

Splenocytes from wt or Polμ^−/−^ animals were cultured in RPMI 1640 medium (Biowhitaker) supplemented with 10% FCS, non-essential amino-acids, 2 mM L-glutamine, 1 mM sodium pyruvate, 10 U/mL penicillin/estreptomycin, 10 µg/ml LPS (Sigma) and 10 ng/ml IL-4 (Preprotek Inc.). After 72 hours, cultures were gamma irradiated at 8 Gy. Cells were collected after 1, 3 and 6 hours; cell were collected by centrifugation and processed for protein extraction.

### Proliferation Assays

To evaluate proliferation during stimulation with SCF and IL-3, bone marrow cells (10^6^) were seeded in Iscove's modified Dulbecco medium (IMDM) supplemented with 10% fetal calf serum (Gibco-BRL), 10% conditioned medium from IL-3-producing WEHI-3B cells, and 50 ng/ml human SCF (Stem Cell Technologies). For long-term bone marrow cultures (LTBMC), whole bone marrow suspensions were seeded in Methocult 5300 (Stem Cell Technologies) with 10^−6^ M hydrocortisone (Sigma), and incubated (33°C, 5% CO_2_) for 1–4 weeks as indicated, with a weekly culture medium change.

Mouse embryonic fibroblasts (MEF) were grown in Dulbecco's Modified Eagle's Medium (DMEM) supplemented with 10% FCS, 2 mM L-glutamine, 10 mM HEPES, 1 mM sodium pyruvate, non-essential amino acids, and 50 µg/ml gentamycin (all from BioWhittaker). Cell cultures were maintained at 37°C, 95% humidity and 5% CO_2_.

For MEF growth assays, embryos were minced with a razor blade and incubated in 0.25% trypsin, 0.1% EDTA for 30 min at 37°C. The cell suspension was plated in DMEM, 10% FCS containing antibiotics. Confluent cultures were split and passage cultures plated at 1×10^6^ cells on 10-cm tissue culture plates (Falcon). Cells were trypsin passaged every 3–4 days. Cumulative cell growth was calculated with the formula PD = log(nf−ni)log2, where PD is population doubling and ni and nf are the initial and final number of cells.

### Clonogenic Assays

Cells were cultured in the appropriate differentiation medium: Methocult M3630 for PreB colonies; M3534 for CFU-G+M colonies; and M3231 with 10% WEHI-3B-conditioned medium, SCF (50 ng/ml) and erythropoietin (6 U/ml) for BFU-E colonies (all from Stem Cell Technologies). Colonies were counted 7 days after seeding. When indicated, colony area was measured by analysis of photomicrographs with ImageJ (http://rsb.info.nih.gov/ij/).

### Competitive Repopulation Assay

Bone marrow mononuclear cells (BMNC) were recovered from male wt or Polμ^−/−^ C57BL/6 mice (which express the CD45.2 surface antigen in hematopoietic cells) as described above. Cells were prepared from four animals per genotype. The Polμ^−/−^ or wt cells (2×10^6^) were mixed with 2×10^6^ competitor bone marrow mononuclear cells from female B6.SJL mice (which express the CD45.1 surface antigen in hematopoietic cells). The mix was injected into the tail veins of female previously irradiated B6.SJL recipients (see below). Repopulation was monitored over 4 months post-transplant by FCM to detect the percentage of CD45.1^+^ and CD45.2^+^ cells in peripheral blood. Four months post-transplant, animals were sacrificed and the relative population of CD45.1^+^ and CD45.2^+^ cells was analyzed in all hematopoietic organs. In parallel, the number of male cells was assessed by Y-chromosome FISH [27]- and Y chromosome-specific PCR [28].

The number of repopulating units (RU) was determined as described [29]. Briefly, number of donor RU = (percentage of donor cells×number of competitor RU)/(100-percent of donor cells). We have assumed that 1×10^5^ competitor cells contain 1 RU.

### Comet Assay

Comet assays were performed with the Cometassay kit (Trevigen). Comet images were obtained for at least 50 cells per condition and analyzed with TriTek CometScore (http://www.tritekcorp.com).

### DNA Damage Assays

For γ-irradiation survival assays of myeloid clonogenic forming units (CFU-C), bone marrow cells were seeded as for clonogenic assays and immediately irradiated (0–8 Gy) in a Cesium Mark1 irradiator (Shepherd Associates). The number of surviving CFU-C colonies was scored 7 days post-irradiation. For γ-irradiation survival assays with MEF, 3×10^2^ cells were seeded in 10 cm^2^ culture plates (Corning Inc.) and irradiated (0–8 Gy) after 24 hours; Crystal Violet stained colonies were counted after 7 days.

### Whole-Body γ-Irradiation

To evaluate differential sensitivity to irradiation, mice were exposed to γ-irradiation (4–10 Gy, single dose) and closely monitored throughout the experimentation period. Animals were killed at the first appearance of signs of poor health. Recipients of bone marrow transplants were pre-irradiated with a total dose of 10.2 Gy (two doses of 5.1 Gy, 3 h apart). For *in vivo* DNA damage experiments, mice were irradiated with a single dose of 5Gy and killed after 1, 3 or 6 hours. All irradiations were carried out in a Cesium Mark1 irradiator (Shepherd Associates).

### Immunofluorescence Microscopy

Cell suspensions were deposited on superfrost slides (Menzel-Glaser) for 5 min. Adhered cells were fixed in 4% paraformaldehyde (20 min, 4°C), washed three times in PBS containing 0.1% Tween (PBST) and blocked (1 h) in PBST/10% BSA. Preparations were incubated overnight with anti-phosphorylated γ-H2AX antibody (Upstate) in PBST/1% BSA. After washing with PBST and staining with secondary antibody (40 min), preparations were washed and mounted, with DAPI, in VectaShield mounting medium (Vector).

Confocal images were acquired on a Fluoview FV1000 Olympus microscope using Fluoview version 1.4 acquisition software. TIFF images were analyzed with Image J. Spleen and bone marrow populations were scored for the number of γ-H2AX positive cells and the number of γ-H2AX foci per nuclei.

### Chromosome Instability Analysis

Nine-month-old wt or Polμ^−/−^ mice were irradiated (5 Gy) and bone marrow cell suspensions prepared after a 6 h recovery period, to allow time for *in vivo* DNA repair. Cells (2×10^6^/ml) were cultured in Myelocult 5300 medium (Stem Cell) supplemented with 20% FCS, 10% WEHI cell-conditioned medium (IL-3) and antibiotics. Metaphase spreads were prepared four days after irradiation.

For telomere *in situ* hybridization (Tel-FISH), metaphase cells were hybridized with a telomeric Cy3- or FITC-labeled PNA-(CCCTAA)_4_ probe (Applied Biosystems) essentially as described [30], except that post-hybridization washes (3×10 min) were performed in PBST at 50°C. FISH images were captured with a Nikon 80I microscope fitted with a 100×1.3 NA planfluor objective and an Olympus DP digital camera. Between 40 and 80 metaphase spreads were scored for chromosomal aberrations (chromosome and chromatid breaks, dicentrics, rings and Robertsonian-like chromosomes: see examples in Figure S5).

### Western Blot and Histone Extraction

For non-histone proteins, extracts were prepared with RIPA lysis buffer (150 mM NaCl, 1% NP-40, 0.5% deoxycholate, 0.1% SDS, 50 mM Tris, pH 8.0). Acidic extraction of histones was carried out as follows. Tissue or pelleted cells were mechanically disaggregated in 0.25 M HCl, incubated at 4°C with agitation for 14 h, centrifuged (13000 rpm, 10 min, 4°C), and further incubated for 4 h in 8 volumes of acetone. After centrifugation (5000 rpm, 5 min), pellets were washed with acetone and dried in a SpeedVac. Extracts were incubated in 0.25 M HCl for 2 hours at 4°C and centrifuged at 13000 rpm prior to quantification and loading on SDS-PAGE gels. After transfer to PDVF membranes, blots were probed with the following antibodies: mouse monoclonal anti-p21 (Santa Cruz Biotechnology, sc-6246, 1∶1000), mouse monoclonal anti-beta actin (Abcam, ab8226-100, 1∶5000), rabbit polyclonal anti-H2AX-P (Upstate, 07-164, 1∶1000) and rabbit polyclonal anti-H3 (Abcam, ab8226-100, 1∶5000).

### ROS Measurement

Wt or Polμ^−/−^ bone marrow cells were irradiated at 4 or 8 Gy. Cells (1×10^6^) were incubated (1 h) with 5 µM DCFDA (Molecular probes) in reduced-serum OPTIMEM (Gibco) and analyzed by FCM. DCFDA fluorescence was analyzed by measuring the mean Fl-1 fluorescence intensity of at least 10000 cells from three animals.

### Statistical Analysis

Unless indicated all analyses were by Student's t test. *:p<0.05; **:p<0.01; ***:p<0.001.

## Results

**Figure 1:**
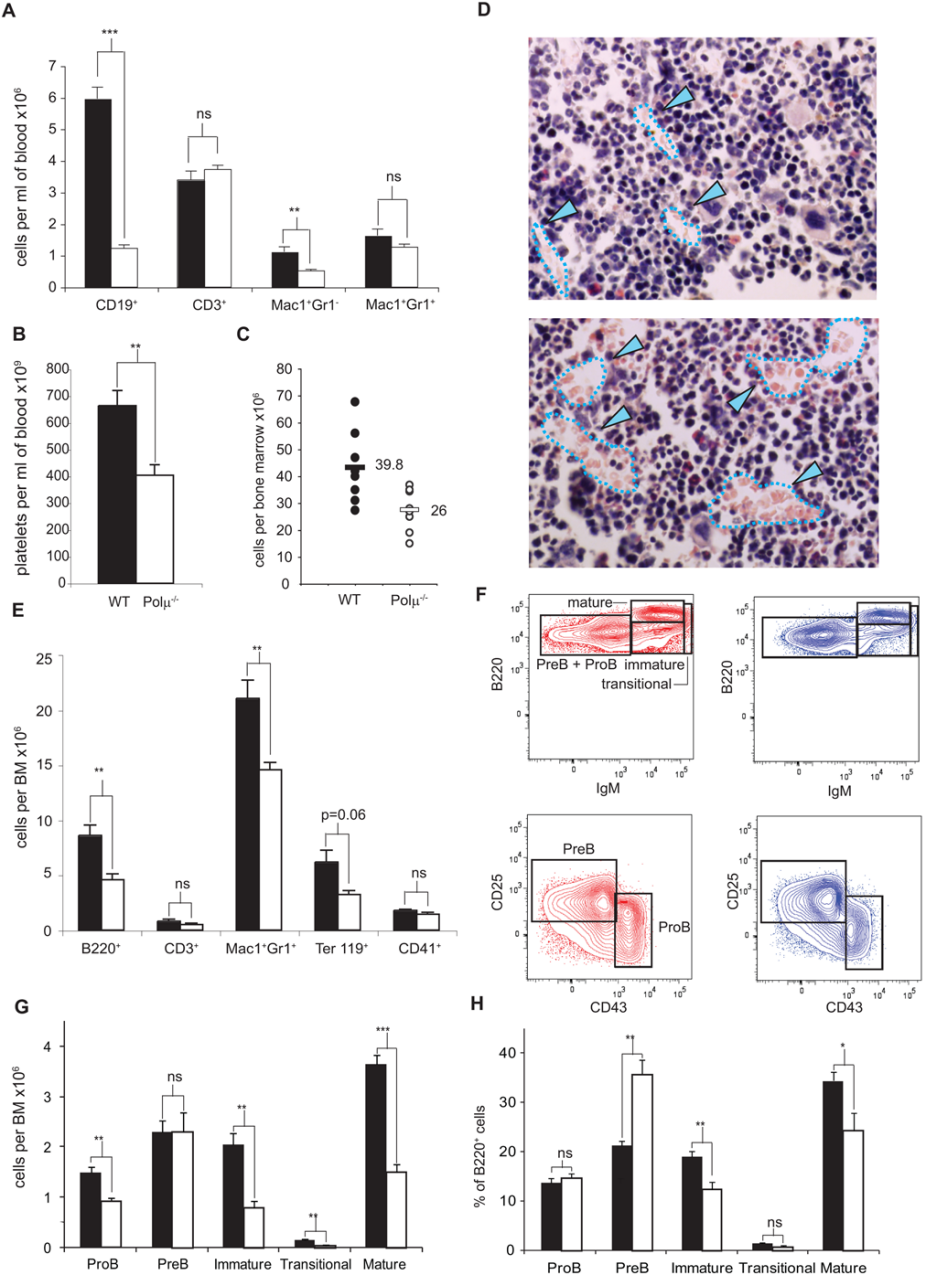
Blood and bone marrow cell profiles are altered and bone marrow cell numbers reduced in Polμ^−/−^ mice. A. Blood cell populations in wildtype (WT: *n* = 6–12; solid bars) and Polμ^−/−^ mice (*n* = 7–13; open bars). CD19, B lymphocytes; CD3, T lymphocytes; Mac1^+^Gr1^−^, monocytes; and Mac1^+^Gr1^+^, neutrophils. B. Representative experiment showing platelet numbers in WT (solid bar; *n* = 6) and Polμ^−/−^ mice (open bar; *n* = 16). C. Representative experiment showing distribution of bone marrow (BM) populations (two femurs per mouse) in WT (closed circles; *n* = 4–7) and Polμ^−/−^ mice (open circles; *n* = 4–8). D. Histological sections of Polμ^−/−^ and WT BM; endothelial sinusoids are delineated by a dashed line (blue) and marked by blue arrowheads. E. BM cell population analysis by flow cytometry (WT, solid bars; *n* = 7; Polμ^−/−^, open bars; *n* = 8), showing B220 (B cell) and CD3 (T cell), Mac1Gr1 (myelomonocytic), Ter119 (erythroid), and CD41 (megakaryocytic) lineages. F. Representative flow cytometry plots of B cell differentiation analysis in the bone marrow of WT (red plots) or Polμ^−/−^ (blue plots) C57BL/6 mice. PreB+ProB cells were further analyzed according to CD25 and CD43 expression to distinguish between PreB and ProB cells. G. Cell number per bone marrow (2 tibias, 2 femur) of the B cell subsets analyzed in F; *n* = 8. H. Frequency (percentage of total B cell population) of the B cell subsets analyzed in F; *n* = 8. Results are pooled data from two independent experiments. Data are means+/−SEM. *: p<0.05; **: p<0.01; ***: p<0.001.

Polμ is widely expressed, but is especially abundant in lympohematopoietic tissues and cells [11],[14]. Polμ is known to participate in DSB repair reactions *in vitro* [15]–[17], but the only *in vivo* role assigned so far is in V_κ_-J_κ_ recombination during B cell development [18]. To investigate whether Polμ has a function in other hematopoietic lineages, we first studied peripheral blood (PB) cell populations in Polμ^−/−^ mice. Concurring with the reduction in B cell numbers associated with abnormal V_κ_-J_κ_ recombination, [18], the B cell count in Polμ^−/−^ mice was 4.9-fold (p<0.001) lower than in wt controls (Figure 1A; CD19^+^ cells). Monocyte numbers were also reduced (2.2-fold, p<0.01, Figure 1A; Mac1^+^ Gr1^−^ cells), as were neutrophil numbers, though here the difference was not statistically significant (p<0.09, Figure 1A; Mac1^+^Gr1^+^ cells). The only cell type analyzed that was apparently unaffected was the T cell lineage (CD3^+^ cells). Hematological analysis revealed moderate thrombocytopenia, with 403×10^9^ platelets/ml in Polμ^−/−^ mice, compared with 663×10^9^ platelets/ml in wt counterparts (p<0.01, Figure 1B); this was associated with increased bleeding times (Figure S1) Thus peripheral blood populations other than B lymphocytes are altered in Polμ^−/−^ mice. To assess whether the defective blood population profile originated in bone marrow (BM), we compared BM cell numbers between wt and Polμ^−/−^ mice. Our results show that BM cellularity was 1.5-fold lower in Polμ^−/−^ mice (p<0.001, Figure 1C). Immunohistochemistry confirmed that Polμ^−/−^ BM contained fewer cells than wt BM, as shown by the dilated endothelial bone marrow sinusoids (Figure 1D). Therefore the most likely cause of the thrombocytopenia and reduced myeloid cell numbers in peripheral blood in Polμ^−/−^ mice is a defect in BM hematopoiesis.

To investigate the function of Polμ during hematopoiesis, we analyzed wt and Polμ^−/−^ (C57BL/6) BM cells by flow cytometry (FCM). The B lymphoid and myeloid compartments were significantly reduced in Polμ^−/−^ BM (p<0.05), and there was a more moderate reduction in erythroid cell numbers (Figure 1E). To determine whether the reduction in B cell numbers was exclusively due to the previously described V_κ_-J_κ_ defect [18], we used FCM to analyze B cell development in the bone marrow of Polμ^−/−^ or wt C57BL/6 mice (see Figure 1F for representative plots and gating strategies). With the exception of PreB cells (B220^+^IgM^−^CD43^−^CD25^+^ cells), all BM B cell populations were significantly reduced in Polμ^−/−^ BM. PreB cell frequency was increased in Polμ^−/−^ BM (Figure 1H), indicating an accumulation at the PreB stage, probably due to a blockade at the PreB-to-immature (IgM^+^) cell transition. Similar results were obtained with Polμ^−/−^ animals in the mixed background (not shown). These results suggest that low B cell numbers in Polμ^−/−^ mice are not only the result of deficient V_κ_-J_κ_ recombination but also reflect altered hematopoiesis prior the specific defect during V_κ_-J_κ_ recombination described previously [18], suggesting that Polμ deficiency affects most hematopoietic lineages in bone marrow.

**Figure 2:**
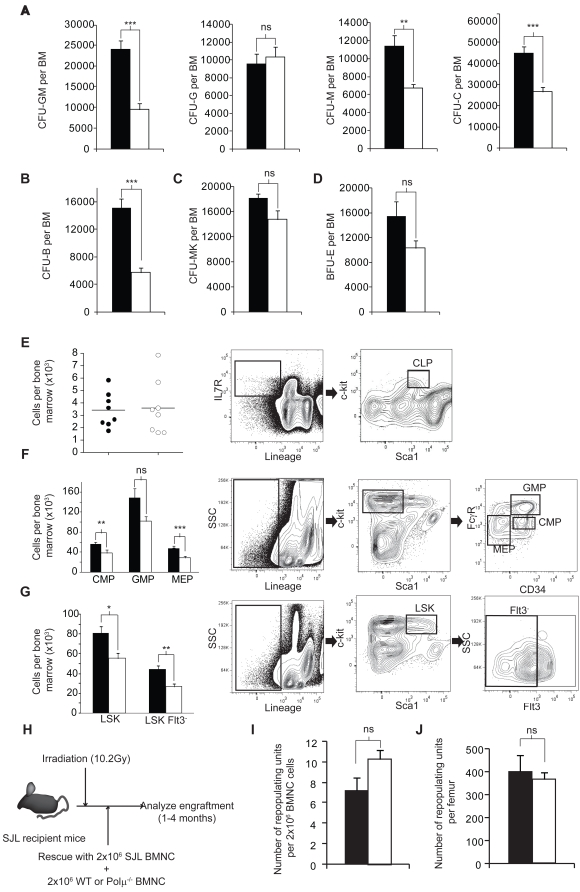
Hematopoietic progenitor and stem cell numbers are reduced in Polμ^−/−^ mice. A. Number of myeloid progenitors (colony forming units) per bone marrow determined in clonogenic assays in WT (solid bars, *n* = 9–11) and Polμ^−/−^ (open bars, *n* = 9–11) mice. CFU-GM (granulomonocytic cells), CFU-G (granulocytes), CFU-M (monocytes) and CFU-C (sum of CFU-GM, CFU-G and CFU-M cells). B. Number of CFU-PreB progenitors. C. Megakaryocyte colony-forming units (CFU-Mk). D. Number of erythroid burst-forming units (BFU-E). BFU-E were scored only if hemoglobin was evident within the colony. E. Flow cytometric determination of CLP (common lymphoid progenitors) in bone marrow of WT (closed circles, *n* = 8) or Polμ^−/−^ (open circles, *n* = 8) C57BL/6 mice (left panel). F. Flow cytometric determination of bone marrow CMP (common myeloid progenitors), myelomonocytic progenitors (GMP) and megakaryocyte/erythroid progenitors (MEP) from WT (solid bar, *n* = 8) and Polμ^−/−^ (open bar, *n* = 8) C57BL/6 mice (left panel). G. Flow cytometric determination of HSC in the bone marrow of the same mice analyzed in F; WT (solid bar, *n* = 8) and Polμ^−/−^ (open bar, *n* = 8) C57BL/6 mice (left panel). Gating strategies for the analysis in E–G are indicated in the plots to the right. H. Experimental design for the competitive bone marrow transplantation assay. I. Frequency of competing repopulating units (RU) in the bone marrow of C57BL/6 wild-type (*n* = 4) or Polμ^−/−^ mice (*n* = 4). J. Number of competing repopulating units (RU) per bone marrow of the mice analyzed in I. Data are means+/−SEM. *: p<0.05; **: p<0.01; ***: p<0.001.

Hematopoiesis is a clonal process in which immature progenitors give rise to more committed progenitors. We therefore used colony forming unit (CFU) assays to determine total progenitor numbers in wt and Polμ^−/−^ BM (Figure 2A–D). The numbers of PreB and myeloid progenitors (CFU-PreB and CFU-C) in Polμ^−/−^mice were 2.2- and 1.3-fold lower than in wt (p<0.001, Figure 2A,B). Megakaryocytic and erythroid progenitors (CFU-Mk and BFU-E) were also affected, though to a lesser extent (Figure 2C,D). Importantly, whereas granulomonocyte and monocyte progenitors (CFU-GM and CFU-M) were reduced in Polμ^−/−^ BM (correlating with the peripheral blood monocyte deficiency), granulocyte progenitors (CFU-G) were not, thus explaining the absence of neutropenia (Figure 1A). These results show that Polμ deficient mice have lowered numbers of committed progenitors and suggest that lack of Polμ differentially affects lineage-specific progenitors.

To examine this in more depth, we next quantified hematopoietic lineage precursors by FCM. The populations measured were common lymphoid progenitors (CLP) [31], defined as Lin^−^IL7R^−^Sca1^+^c-kit^+^ cells, (Figure 2E); common myeloid progenitors (CMP), myelomonocytic progenitors (GMP) and megakaryocyte/erythroid progenitors (MEP) [32] (Figure 2F); hematopoietic stem cells were defined as Flt3^−^ cells after gating on Lineage^−^c-kit^+^Sca1^+^ [33] (Figure 2G). Although FCM analysis revealed that the frequency of these populations was little altered in Polμ^−/−^ mice (Table S1), myeloid progenitors and stem cells were less abundant in the BM of Polμ^−/−^ mice and some populations were more severely affected (Figure 2E–G); for example, HSC numbers were 39% lower in Polμ^−/−^ BM (p<0.01). We then analyzed engraftment potential of Polμ^−/−^ HSC in a competitive repopulation study [29]. In this experiment 2×10^6^ BMNC (bone marrow nucleated cells) from male wt or Polμ^−/−^ C57BL/6 mice were mixed with 2×10^6^ competing cells from female B6.SJL mice (1∶1), and the mixed population transplanted into lethally irradiated female B6.SJL recipients (see Figure 2H for experimental design). The percentage of donor and competitor cells was determined 16 weeks later and used to calculate the number of repopulating units (RU) in the donor BM. No differences were detected in either the frequency of (Figure 2I) or absolute (Figure 2J) RU numbers between wt and Polμ^−/−^ bone marrow. These results indicate that although Polμ deficiency reduces the numbers of most hematopoietic progenitors and stem cell populations the defect can be rescued (at least partially), by transplantation into a healthy stroma (Figure 2I). Additionally, clonogenic analysis detected very few progenitors in Polμ^−/−^ spleen (Figure S2), indicating that Polμ^−/−^ mice do not compensate for the BM deficit with extramedullary hematopoiesis.

**Figure 3:**
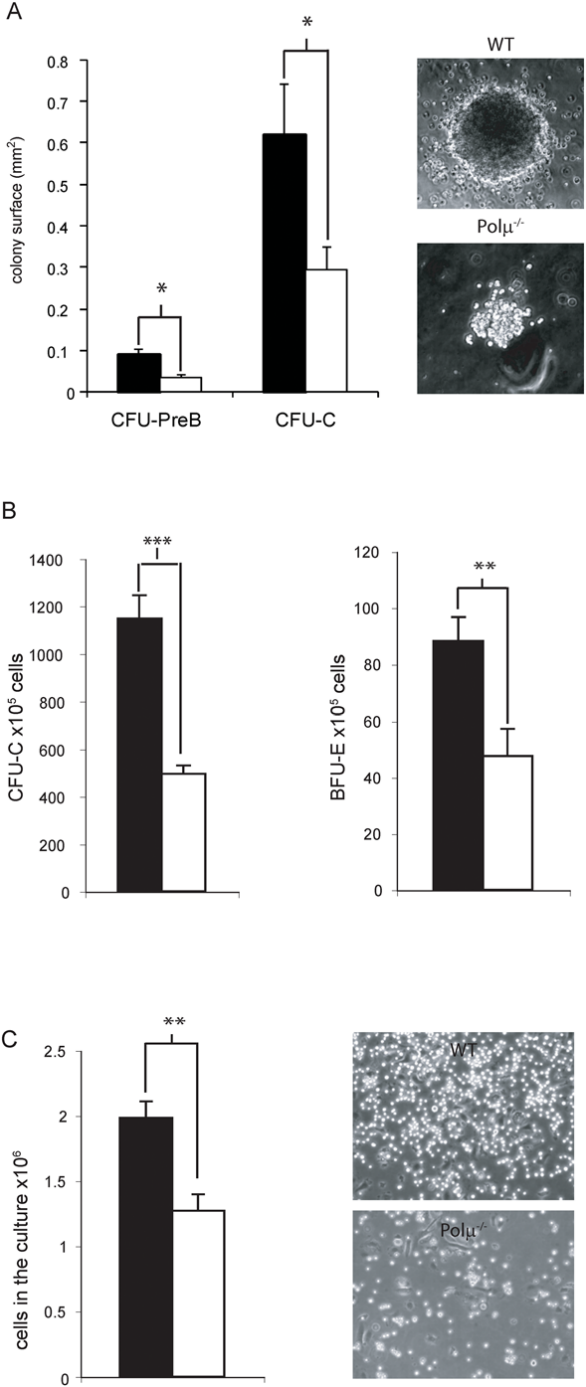
Polμ deficiency reduces the proliferation potential of hematopoietic progenitors. A. Left panel: estimated size (planar surface) of CFU-PreB and CFU-C colonies derived from WT BM (solid bar) and Polμ^−/−^ BM (open bar). Right panel: Micrographs of representative CFU-PreB colonies derived from WT and Polμ^−/−^ BM, showing the reduced colony size of Polμ^−/−^ colonies. B. Numbers of myeloid (left) and erythroid (right) progenitors recovered after 4 days expansion of WT BM (solid bars, (*n* = 7) and Polμ^−/−^ BM (open bars, (*n* = 7) in IL3 and SCF supplemented medium. C. Left: Absolute cell numbers, per culture, recovered after a 2 week expansion of WT (solid, (*n* = 5) and Polμ^−/−^ (open, (*n* = 4) long-term bone marrow cell cultures (LTBMC). Right: representative micrographs of stroma generated in WT and Polμ^−/−^ LTBMC (3 weeks). Data are means+/−SEM. *: p<0.05; **: p<0.01; ***: p<0.001.

The low progenitor numbers in Polμ^−/−^ mice could result either from impaired differentiation or from defective progenitor proliferation or self renewal capacity. To investigate this, we examined the proliferation of Polμ^−/−^ hematopoietic stem and progenitor cells *in vitro*. In clonogenic differentiation assays, colonies of Polμ^−/−^ progenitors were significantly smaller (50% smaller for CFU-C, p<0.05; 60% smaller for CFU-PreB, p<0.05) and contained fewer cells than wt colonies, indicating defective self-renewal or proliferative capacity (Figure 3A). This was confirmed by growing BMNC in conditions that promote myeloid progenitor expansion (see methods). After 4 days, wt BMNC cultures contained 2.3-fold more myeloid progenitors (p<0.001) and 2.8-fold more erythroid progenitors (p<0.01) than Polμ^−/−^ cultures (Figure 3B). We then assayed BMNC in long-term bone marrow cell cultures (LTBMC, see methods). Two weeks after initiation, Polμ^−/−^ cultures contained 35% fewer cells than wt (p<0.01, Figure 3C). Cell cycle analysis revealed an increased incidence of cell death in Polμ^−/−^ cultures (Figure S3). Together with the bone marrow reconstitution experiments, these results suggest that Polμ^−/−^ bone marrow stromal cells fail to efficiently support hematopoiesis and that Polμ^−/−^ hematopoietic progenitor numbers do not expand in vitro as efficiently as WT progenitors.

**Figure 4:**
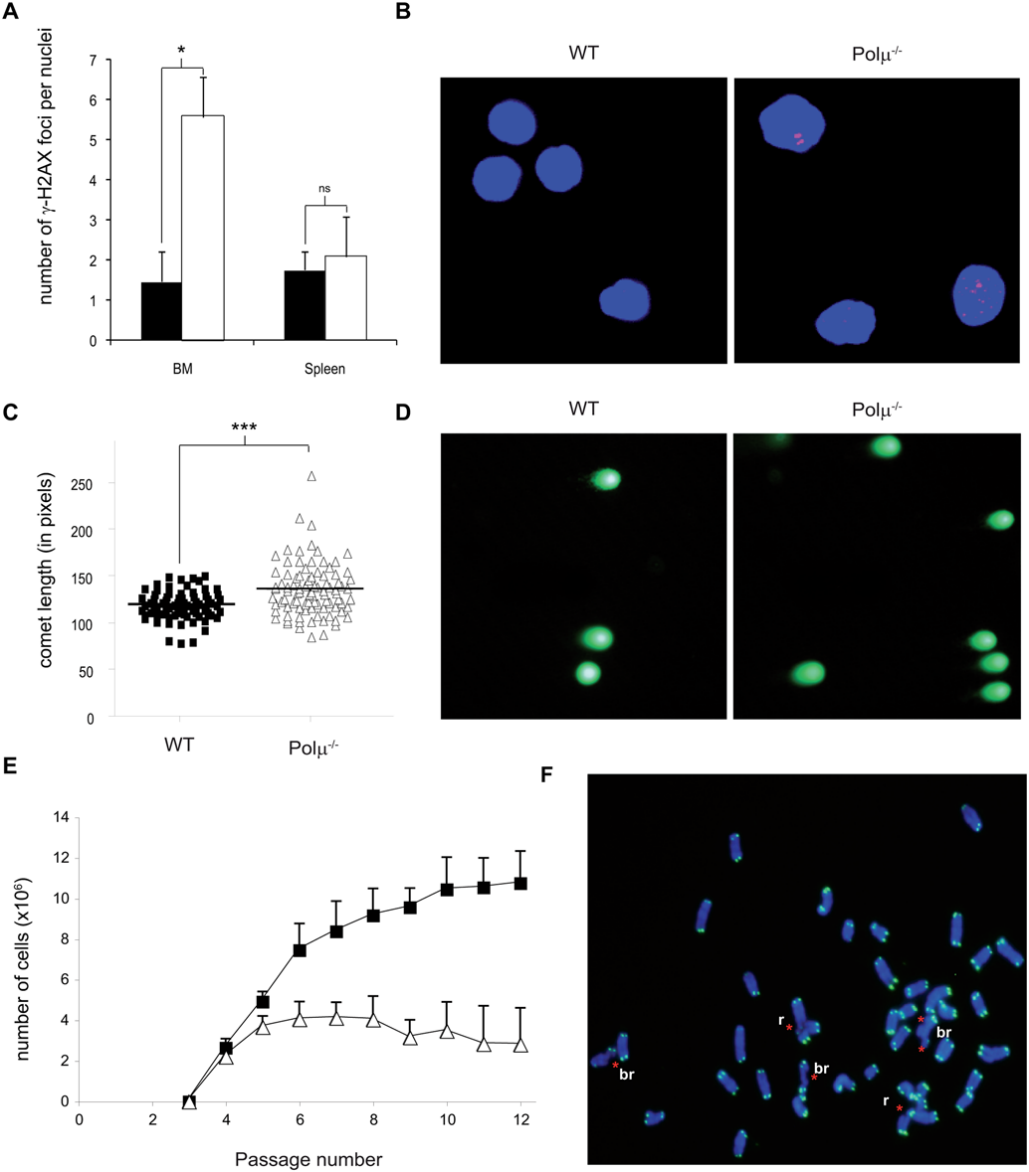
DSB repair is impaired in Polμ^−/−^ mice. A. Numbers of γ-H2AX foci staining per nuclei in WT (solid bar) or Polμ^−/−^ (open bar) BM and spleen cells. B. Representative confocal images showing γ-H2AX foci (Red, Cy3) in WT and Polμ^−/−^ BM cells (blue staining, DAPI). C. Dot plots showing the comet length of WT (closed squares) and Polμ^−/−^ (open triangles) bone marrow cells. The comet assay was performed in neutral conditions (1×TBE) to assess the relative levels of DNA double strand breakage (DSB). D. Representative fluorescence images of comet assay gels from C. E. Reduced proliferation capacity and premature senescence of cultured Polμ^−/−^ MEF. The 3T3 growth assays show the cumulative increase cell number versus passage in primary (passage 3 at the start of the experiment) WT (closed squares) and Polμ^−/−^ (open triangles) cells. Note that WT MEF enter senescence around division 10, but Polμ^−/−^ MEF stop proliferating by division 4. F. Representative image of a Polμ^−/−^ MEF metaphase spread stained by TEL-FISH. The telomeres are stained with a FITC-labeled (Green) PNA probe and chromosomes are counterstained with DAPI (blue). Asterisks indicate specific chromosomal defects: br (break), r (radial configuration). All data are means+/−SEM. *: p<0.05; **: p<0.01; ***: p<0.001.

One possible cause of reduced Polμ^−/−^ hematopoietic precursors numbers is impaired DSB repair. To test this, we stained nucleated cells from bone marrow and spleen for phosphorylated γ-H2AX, a marker of DNA double strand breaks [34]. Compared with wt, Polμ^−/−^ bone marrow had a significantly higher number of DSB per nucleus (3.8 fold, p<0.05; Figure 4 A,B); in Polμ^−/−^ spleen cells (mostly non-proliferating) a smaller increase was observed that did not reach significance (Figure 4A). This suggests that Polμ is required for DSB repair in cycling cells. We confirmed the higher DSB incidence in Polμ^−/−^ bone marrow cells by comet assay, which revealed significantly longer comet tails in Polμ^−/−^ BM cells (p<0.001; Figure 4C,D).

Mouse embryonic fibroblasts (MEF) derived from mice deficient in NHEJ proteins show a highly reduced proliferative capacity compared to wt controls [35]–[37]. To assess the roles of Polμ in DNA repair and proliferation in other tissues, we analyzed wt and Polμ^−/−^ MEF. Consistent with the proposed role of Polμ in NHEJ, our results show that Polμ^−/−^ MEF showed reduced growth after long-term culture and they enter senescence prematurely (Figure 4E). In addition, primary Polμ^−/−^ MEF were genomically unstable compared with wt MEF. In particular, Polμ^−/−^ MEF showed a 4-fold increase in chromosomal aberrations, with a striking 16-fold increase in the level of radial configurations and a 4.5-fold increase in chromosomal breaks, signatures of deficient DNA repair and increased radiosensitivity (Table 1 and Figure 4F; see Figure S5 for examples of chromosome aberrations). These results demonstrate that lack of Polμ results in DSB accumulation in bone marrow and probably in connective tissue. This suggests that proliferation deficiency by Polμ^−/−^ cells is due to accumulation of unrepaired DSB or a general delay in DSB repair, which may lead to cell cycle arrest and cell death [38].

**Table 1:** Quantification of chromosomal aberrations analyzed by Telomere FISH in WT and Polμ^−/−^ BM cells and MEF.

Genotype (IR Gy)	# of meta. analyzed	Robert. type fusions (N)	Dicentrics (N)	Rings (N)	Radials (N)	Breaks (N)	Total aberr. (N)
**WT BM (0 Gy)**	50	1 (2)	0 (0)	1 (2)	0 (0)	11 (22)	13 (26)
**Polμ^−/−^ BM (0 Gy)**	49	1 (2)	0 (0)	1 (2)	0 (0)	16 (32,6)	18 (36,7)
**WT BM (5 Gy)**	64	6 (9,4)	37 (57,8)	1 (1,6)	2 (3,1)	187 (292,2)	233 (364,1)
**Polμ^−/−^ BM (5 Gy)**	65	6 (9,2)	169 (260)	10 (15,4)	10 (15,4)	521 (801,5)	716 (1101)
**WT MEF (0 Gy)**	87	2 (2,3)	1 (1,1)	1 (1,1)	0 (0)	42 (48,3)	46 (52,9)
**Polμ^−/−^ MEF (0 Gy)**	65	3 (4,6)	1 (1,5)	2 (3,1)	16 (24,6)	144 (221,5)	166 (255,4)
**WT MEF (5Gy)**	48	5 (10,4)	14 (29,2)	5 (10,4)	5 (10,4)	344 (716,7)	373 (777,1)
**Polμ^−/−^ MEF (5 Gy)**	42	9 (21,4)	35 (83,3)	7 (16,7)	10 (23,8)	401 (954,8)	462 (1100)

After irradiation, mice were maintained for 6 h to allow *in vivo* DNA repair. Metaphase spreads of BM cells were prepared 4 days post irradiation. Metaphase spreads of primary (passage 6) MEF were prepared 72 h post irradiation. Data in parentheses (N) are the number of aberrations observed per 100 cells analyzed.

**Figure 5:**
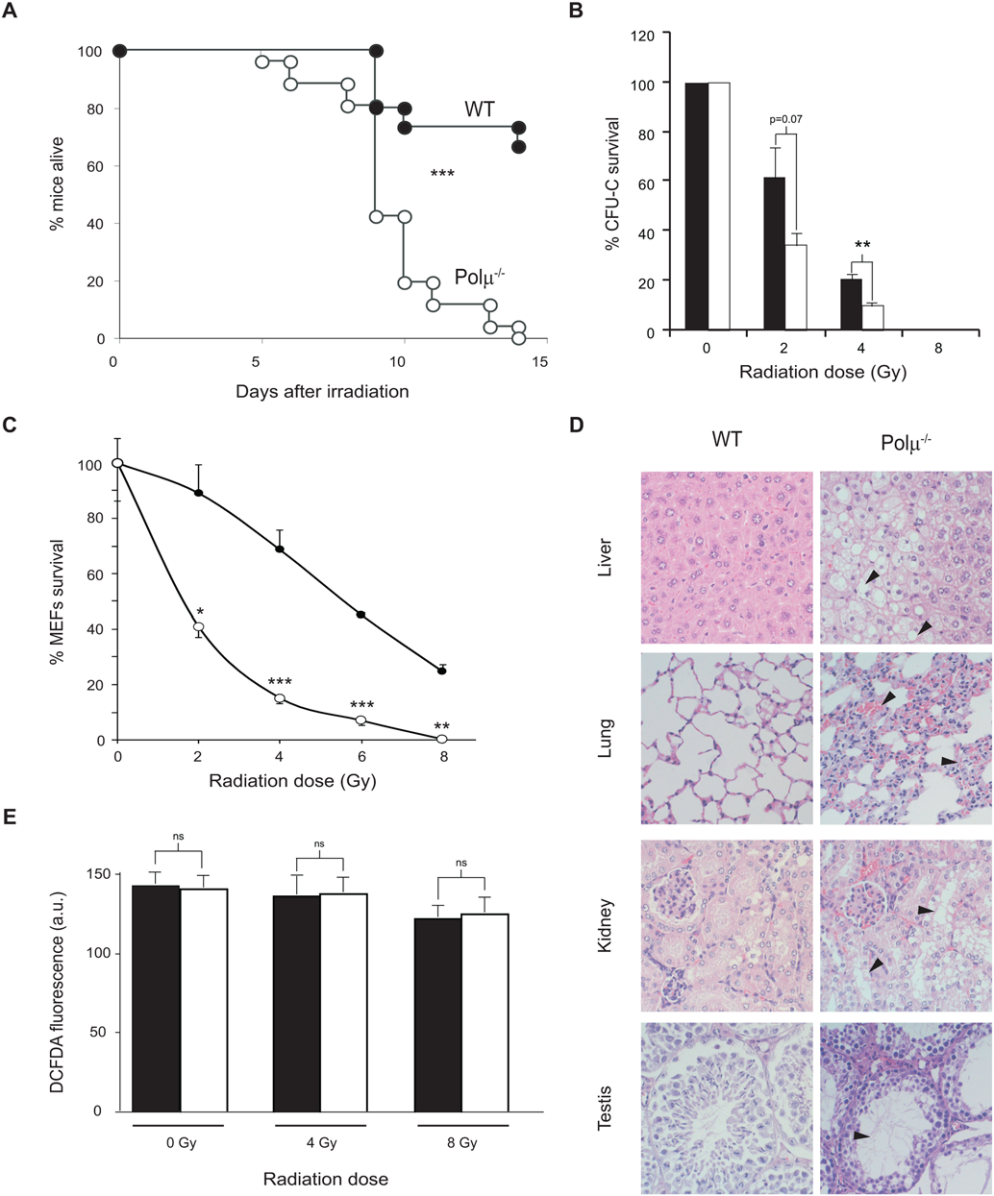
Polμ^−/−^ mice show increased radiosensitivity. A. Survival of Polμ^−/−^ (open circles) and WT mice (closed circles) after whole-body γ-irradiation (9 Gy); *n* = 15. B. Percentage survival by γ-irradiated WT (solid bars) and Polμ^−/−^ (open bars) bone marrow CFU-C progenitors; *n* = 4 mice analyzed in duplicate assays. C. Percentage survival by γ-irradiated WT (closed circles) and Polμ^−/−^ (open circles) mouse embryonic fibroblasts (MEF); The figure shows one experiment with each point assayed in quadruplicate. D. Photomicrographs of formalin-fixed, paraffin-embedded, hematoxylin-eosin-stained sections of liver, lung, kidney and testis from irradiated WT and Polμ^−/−^ mice. Note extensive damage (arrowheads) in Polμ^−/−^ tissues: vacuolar degeneration (liver); inflammation and hemorrhaging (lung) and tubular degeneration (kidney and testis). E. Flow cytometry ROS measurements by DCFDA fluorescence in irradiated and mock-irradiated WT (solid bars) and Polμ^−/−^ bone marrow cells (open bars); *n* = 3. There was no significant difference in ROS levels between WT and Polμ^−/−^ cells under basal conditions or upon irradiation. All data are means+/−SEM. *: p<0.05; **: p<0.01; ***: p<0.001.

To test this *in vivo*, we irradiated Polμ^−/−^ and wt mice (9 Gy) and analyzed survival over time. Fifteen days post-irradiation all Polμ^−/−^ mice were dead, compared with only 40% of wt mice (p<0.001 Log rank test; Figure 5A). Most Polμ^−/−^ mice died between days 9 and 11 (Figure 5A), and hematologic analysis revealed extreme neutropenia (not shown), indicating hematopoietic failure. The impact of Polμ deficiency on the response to irradiation damage is also illustrated by the higher radiosensitivity of Polμ^−/−^ progenitors (Figure 5B; p<0.01). Bone marrow cells from wt and Polμ^−/−^ mice showed G2 accumulation after irradiation (5Gy), suggesting that the G2/M cell cycle checkpoint is functional in Polμ^−/−^ cells (Figure S4A). Western blot detected increased p21 accumulation in irradiated Polμ^−/−^ splenocytes and even in non-irradiated cells (Figure S4B), suggesting activation of the G1 cell cycle checkpoint. Irradiation of Polμ^−/−^ MEF (2–8 Gy) significantly reduced survival compared with similarly treated wt MEF (2 to 123-fold; p<0.05. Figure 5C). Histological analysis of irradiated mice showed vacuolar degeneration in liver (where we detect intense γ-H2AX accumulation after irradiation; Figure S4C), hemorrhage and congestion in the lung, tubular degeneration in kidney, and tubular atrophy in testis (Figure 5D). An alternative explanation for increased tissue damage in irradiated Polμ^−/−^ mice might be increased production of reactive oxygen species (ROS). We therefore measured intracellular ROS levels in irradiated bone marrow cells (4 and 8 Gy) by staining with DCFDA (Di-cloro-fluorescein diacetate: a marker of intracellular peroxides). ROS accumulation was unaffected by Polμ^−/−^ deficiency (Figure 5E), indicating that ROS production does not contribute to the radiosensitivity of Polμ^−/−^ animals or cells. These data thus show that the requirement for Polμ in DNA repair extends to tissues outside the hematopoietic compartment (Figure 5C,D).

**Figure 6:**
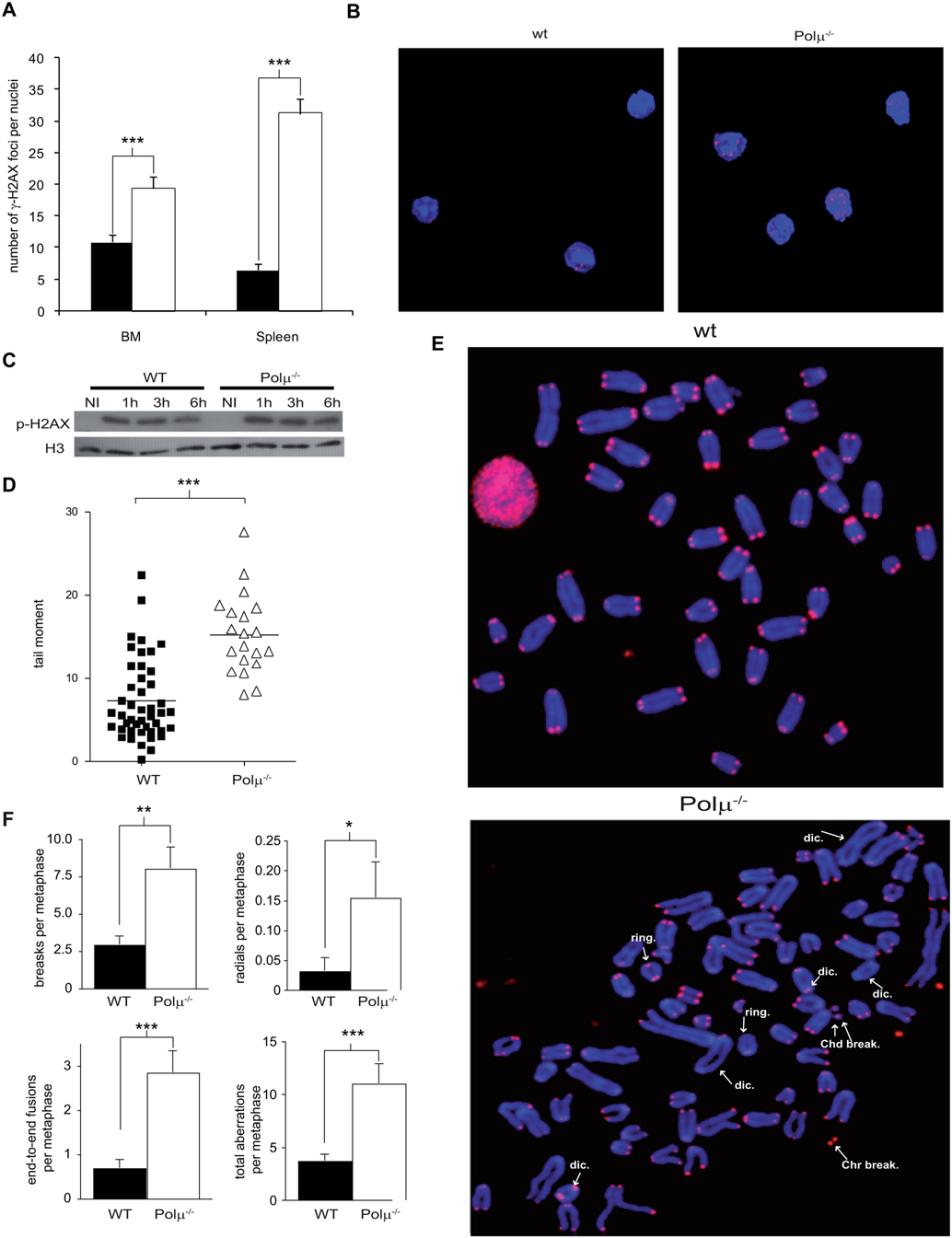
Polμ^−/−^ hematopoietic progenitor cells have a reduced capacity for DSB repair. A. Number of γ-H2AX foci per nucleus in bone marrow and spleen cells from WT (solid bars) and Polμ^−/−^ (open bars) γ-irradiated mice (5Gy); staining was performed 1 hour post-irradiation. B. Representative confocal images showing γ-H2AX foci (Red, Cy3; blue, DAPI) in WT and Polμ^−/−^ bone marrow cells treated as in A. C. Western blot showing amounts of phosphorylated γ-H2AX protein in LPS stimulated wt and Polμ^−/−^ splenocytes after γ-irradiation (8 Gy), and analyzed at different periods (1 h, 3 h, and 6 h) post-irradiation; NI: non-irradiated controls. Histone H3 was used as a loading control. D. Dot plot showing DNA comet tail moment of WT (closed squares) and Polμ^−/−^ (open triangles) bone marrow cells retrieved from irradiated mice (5Gy) after 3 hours of *in vivo* DNA repair. Tail momentum was significantly increased in Polμ^−/−^ cells. E. Representative images of irradiated WT and Polμ^−/−^ bone marrow metaphase cells analyzed by telomere FISH 96 hours after irradiation. Telomeres were hybridized with a Cy-3 labeled PNA Probe (Red) and chromosomes counterstained with DAPI (blue). Arrows indicate structural chromosomal aberrations: Chd and Chr = chromatid and chromosome breaks, respectively; dic. = dicentric chromosomes. F. Number of breaks, radial configurations, end-to-end-fusions, and total aberrations per metaphase in bone marrow cells from WT (closed bars) and Polμ^−/−^ (open bars) irradiated mice. The mice were γ-irradiated (5Gy) and maintained (6 h) to allow *in vivo* DNA repair; BM-cell chromosomal aberrations were detected 4 days post irradiation. All data are means+/−SEM. *: p<0.05; **: p<0.01; ***: p<0.001.

To examine the effect of Polμ deficiency on DNA repair in the hematopoietic system more closely, we analyzed the frequency of phosphorylated γ-H2AX foci in bone marrow and spleen after whole animal γ-irradiation (5Gy). In both genotypes 91% of all BM cells were γ-H2AX^+^, showing that these cells are more susceptible to γ-irradiation. In contrast, the proportion of phospho-γ-H2AX positive cells in Polμ^−/−^ splenocytes (95%) was significantly higher than in wt cells (27%; p<0.001). Further, the number of γ-H2AX foci per cell was significantly increased in Polμ^−/−^ cells compared with wt (1.7-fold for BM cells and 4.8-fold for splenocytes, p<0.001; Figure 6A–B). Changes in γ-H2AX phosphorylation were confirmed by western blot of γ-irradiated (8 Gy) spleen B cells; as predicted, Polμ^−/−^ cells show higher amounts of phospho-γ-H2AX, and these levels are sustained for longer (Figure 6C). We confirmed reduced DSB repair in Polμ^−/−^ BM cells by comet assay. Before cell extraction, irradiated mice (5Gy) were left to recover for 3 hours to allow *in vivo* DNA repair (see Methods). BM cells from these mice were separated by single cell electrophoresis under alkaline conditions, to measure the relative levels of DNA breaks; comet tail moment was 2-fold higher in Polμ^−/−^ cells compared with wt cells (p<0.001; Figure 6D), indicating reduced DNA repair in Polμ^−/−^ cells.

The effect of the impaired DNA repair on genetic stability was investigated by telomere-directed fluorescent *in situ* hybridization (Tel-FISH) and DAPI staining of control or irradiated (5Gy) wt and Polμ^−/−^ BM metaphase cells. Figure 6E shows representative results from irradiated cells. These experiments detected a higher level of genomic instability in Polμ^−/−^ cells both before and after irradiation. Non-irradiated Polμ^−/−^ samples demonstrated only a 0.5-fold increase in chromosome breaks compared with controls (32.6 vs. 22 breaks per 100 cells). After irradiation, the total number of aberrations was 3-fold higher in Polμ^−/−^ cells (1101 vs. 364 per 100 cells in Polμ^−/−^ and wt; (p<0.0003). Compared with wt, chromosome breaks in irradiated Polμ^−/−^ cells were increased 2.7 fold (p<0.0014), radial configurations 4.8 fold (p<0.05), and end-to-end fusions (dicentrics, Robertsonial-like fusions and rings) 3.5 fold (p<0.0003) (Figure 6F, Table 1). Supplemental Figure 5 shows example images of the chromosomal alterations analyzed. These results confirm that Polμ is required for DSB repair *in vivo* in hematopoietic and non-hematopoietic systems, particularly in response to genotoxic stress.

## Discussion

Polμ is a gap filling, error-prone DNA polymerase that, in association with the NHEJ core machinery, promotes microhomology use during DSB repair [17],[39]. Polμ deficiency does not affect mouse development or generate a severe phenotype in adults [14]. This is probably because its function can be replaced or compensated, at least in part, by other DNA polymerase activities [14],[17]. The most prominent phenotype reported to date in Polμ^−/−^ mice is partial impairment of immunoglobulin V_κ_-J_κ_ recombination [18], associated with alterations in the peripheral B cell compartment [18]. Polμ^−/−^ mice are nonetheless able to mount an almost normal humoral immune response [14],[40].

Here we show that Polμ is required for DSB repair *in vivo* and that this defect confers radiosensitivity, promotes genetic instability and leads to deficient hematopoiesis. Polμ deficiency alters many peripheral blood cell populations, affecting not only the B cell compartment [14], but also the myeloid lineages (Figure 1A). Polμ^−/−^ bone marrow showed a severe reduction (∼40%) in total bone marrow cell numbers (Figure 1C, D). This reduction affects all hematopoietic lineages as demonstrated by flow cytometry (Figure 1). Interestingly, B cell development is affected prior V_κ_-J_κ_ recombination (Figure 1F–H). All committed hematopoietic progenitors were reduced in Polμ^−/−^ BM; the hematopoietic defect was traced back to the hematopoietic stem cell, since FCM revealed a reduction in the number of HSC (Figure 2G). Interestingly, transplant of Polμ^−/−^ bone marrow (together with competitor WT cells) into irradiated recipients rescued (at least partially) the hematopoietic defect, suggesting that Polμ^−/−^ stroma does not completely support hematopoiesis, alternatively, the co-transplantation of hematopoietic wt cells might provide factors that support the development of Polμ^−/−^ HSC. Polμ^−/−^ progenitors do not expand *in vitro* as efficiently as WT controls, suggesting that lack of Polμ results in cell death or reduced proliferation. Taken together these results suggest that hematopoietic cells in the bone marrow of Polμ^−/−^ mice are reduced due to a combination of extrinsic (defective bone marrow stroma) and intrinsic (reduced DNA repair and expansion potential) factors.

We demonstrate that hematopoietic Polμ^−/−^ cells contain more DSB than their wt counterparts, as shown by the increase in the basal level of phospho-γ-H2AX positive cells and comet assay results (Figure 4A–D) and increased basal chromosomal breakage (Table 1). The reduced DSB repair capacity of hematopoietic Polμ^−/−^ cells is more evident after genotoxic insult, demonstrating increased genomic instability (Figure 6; Table 1). Our results also show that Polμ is required for DNA repair in other tissues; including connective tissue (MEF) (Table 1 and Figure 4E–F), liver and kidney (Figure 5B). Lack of Polμ results in radiation-induced cell death at doses that are non-lethal for wt mice (Figure 5A). However, immunohistochemistry failed to demonstrate any gross tissue or organ damage outside the hematopoietic system in non-irradiated Polμ^−/−^ mice (not shown). This suggests that Polμ deficiency may be compensated for or tolerated in organs in which progenitor cell proliferation is slower than in the hematopoietic system or in MEF. Alternatively, it may indicate that - in some tissues - Polμ function is only required under stress conditions (for example, irradiation), which require faster repair. This is further supported by the fact that although most hematopoietic lineages are reduced in Polμ^−/−^ BM, different subsets are affected to different extents. For example although CFU-GM and CFU-M progenitors are reduced in Polμ^−/−^ BM, CFU-G progenitors are not. Another example is the reduction of the HSC population by 39% (p<0.001), while the CLP is unaffected. Whether the less-affected cell types have less DNA damage or use different DNA repair mechanisms remains to be investigated.

To our knowledge this is the first report to show increased radiosensivity in Polμ deficient mice and cells. A previous study reported a relative lack of radiosensivity in Polμ^−/−^ MEF [18]. This contrasts with our data, which strongly suggest that Polμ is necessary for the repair of double strand breaks. Polμ-deficient MEF showed reduced growth curves and increased basal and radiation-induced levels of chromosomal aberrations; phenotypes similar to those previously reported for Ku80^−/−^ and LigIV−/− MEF [30], [35], [36], [37], [41]–[45]. Apart from the different genetic background of the two Polμ^−/−^ mouse models, there are other possible explanations for this discrepancy. First, the gene targeting strategy is different between the two Polμ^−/−^ mouse models. In our model, exons 2 to 4 are deleted, which eliminates the BRCT domain and completely abrogates protein expression in Western blot analysis [14]. In the knockout generated by Bertocci and coworkers, exons 6 to 11 are deleted, eliminating the core of the polymerase activity. Although these mice do not express full-length Polμ mRNA, no information is provided regarding protein expression [18],[40]. This is important, since a truncated Polμ BRCT domain with biological activity may be expressed in this mouse model. An alternative explanation is that the two studies use different experimental procedures to detect radiosensitivity. We γ-irradiated MEF cultures plated at clonogenic dilutions (300 cells in 10 cm^2^ dishes, see Methods) and calculated survival by scoring colonies. In contrast, [18] performed cell survival assays at much higher cell densities and used X-rays as the agent of DNA damage; survival was assessed by trypan blue exclusion. Differences in the sensitivities of these approaches might account for the differences observed in MEF survival in the two models.

The role of DNA repair in hematopoietic homeostasis has become increasingly clear in recent years [20]–[23]. Knockout models of many DNA repair enzymes are characterized by either reduced stem cell function or alterations in specific hematopoietic populations. Moreover, since stem cells are protected from apoptosis [20],[21], probably because of their quiescent state [46], deficiency in DNA repair enzymes allows mutations to accumulate in these cells. Conversely, more committed progenitors, which cycle more rapidly, would be more sensitive to cell cycle arrest or apoptosis, thus explaining the observed reduction in the numbers of progenitor cells in Polμ-deficient mice. Thus the survival of HSC comes at the price of accumulated mutations; two recent reports show that mice deficient in DNA repair maintain expanded stem cell pools, but that these pools have a reduced differentiation potential during aging [20],[21].

Polμ deficiency, as reported for other NHEJ factors such as Ku80 [21] and Ligase IV [20], targets the hematopoietic system and HSC. There are, however, striking differences in the phenotypes observed in Ku80 and LigaseIV mice and Polμ^−/−^ mice. In the first two cases, HSC were unaffected in younger mice but were progressively exhausted during aging. Polμ unlike Ku80 or Ligase IV only participates in a small number of NHEJ reactions that require an error-prone DNA polymerase to generate or promote microhomology pairing between the DNA ends of the DSB [15]–[17] and Polμ^−/−^ mouse has reduced HSC numbers in the bone marrow. These suggest that different components of the NHEJ system will have different roles in hematopoiesis or that some of the DSB generated in hematopoietic cells require Polμ for their complete and efficient repair, and cannot be repaired by compensatory mechanisms with sufficient speed or efficiency to prevent cell death or premature senescence. Additionally, the whole body irradiation experiments presented here show that DSB-repair defects and cell death in Polμ^−/−^ mice are not restricted to the hematopoietic system but are widespread, demonstrating a role for Polμ in DSB repair in other tissues. Our results thus show that Polμ is required for genomic stability and DNA repair, through its participation in DSB repair in hematopoietic stem and progenitor cells as well as in non-hematopoietic cells.

## Supporting Information

Figure S1 Increased bleeding in Polμ^−/−^ mice. Bleeding time (in seconds) of Polμ^−/−^ (open circles) and WT (solid circles) mice in a coagulation assay.
(0.25 MB EPS)

Figure S2 Polμ^−/−^ mice do not show enhanced extramedullary hematopoiesis. Total spleen progenitors (PreB: CFU-PreB, GM: CFU-GM; G: CFU-G; M: CFU-M; see legend to Figure 2 for details ) in Polμ^−/−^ (open bars) and WT mice (solid bars).
(0.22 MB EPS)

Figure S3 Increased cell death in Polμ^−/−^ cultures. Flow cytometry analysis of cell cycle distribution in long term bone marrow cultures derived from Polμ^−/−^ (open bars) and WT mice (solid bars), 4 days after culture initiation.
(0.76 MB EPS)

Figure S4 Cell-cycle arrest in Polμ^−/−^ cells after γ-irradiation. A. Cell-cycle profile of WT and Polμ^−/−^ bone marrow cells in non-irradiated and irradiated (5Gy) animals. Upon irradiation both strains show an accumulation of cells in the G2/M cell-cycle stage (R5 gate), suggesting that the G2/M cell-cycle checkpoint is functional. B. Western blot of non-irradiated (NI) and irradiated (8Gy) wt and Polμ^−/−^ splenocytes collected at different times post irradiation (1 h, 3 h, 6 h). The figure reveals an intense accumulation of p21 in the Polμ^−/−^ cells, suggesting that the G1 cell-cycle checkpoint is functional. Beta-actin was used for normalization and as a loading control. C. Western blot of phosphorylated γ-H2AX content in liver from irradiated (5Gy) wt or Polμ^−/−^ animals. Accumulation of phosphorylated H2AX is evident in the Polμ^−/−^ livers. Histone H3 was used as a loading control.
(1.32 MB EPS)

Figure S5 Examples of chromosomal aberrations detected by Telomere FISH (TEL-FISH) in Polμ^−/−^ bone marrow cells after irradiation. The panels show selected Polμ^−/−^ metaphase chromosomes hybridized to a Cy-3 PNA telomeric probe (Red) and counterstained with DAPI (blue). Since telomeres mark the natural ends of the chromosomes, TEL-FISH significantly improves the resolution of the cytogenetic analysis. Asterisks indicate the sites of individual aberrations. The bottom panel shows an undamaged chromosome, with four telomeres capping each chromatid end.
(1.47 MB EPS)

Table S1 Frequency of hematopoietic progenitor and stem cells in Polμ^−/−^ bone marrow.
(0.01 MB PDF)
