# Supplementary material for: Altered Hematopoiesis in Mice Lacking DNA Polymerase μ Is Due to Inefficient Double-Strand Break Repair
Source: PLoS Genet. 2009 Feb 20;5(2):e1000389. doi: 10.1371/journal.pgen.1000389 (PMC2638008; doi:10.1371/journal.pgen.1000389)
Supplement: Table S1 — Frequency of hematopoietic progenitor and stem cells in Polμ−/− bone marrow. (0.01 MB PDF) [file pgen.1000389.s006.pdf]

GATE	Lin$^-$	LSK	Lin$^-$IL7R$^+$	Lin$^-$Sca1$^-$c-kit$^+$		
% of	*LSK cells*	*HSC*	*CLP*	*CMP*	*GMP*	*MEP*
**WT**	4.03±0.48	55.20±1.52	2.68±0.25	17.32±1.61	43.87±2.9	14.6±1.2
**Polµ$^{-/-}$**	5.35±0.71	48.37±2.44	3.76±0.46	17.26±1.48	47.75±2.24	13.15±0.79

Table S1. Frequency of hematopoietic progenitor and stem cells in Polµ$^{-/-}$ bone marrow. The table shows the percentage (frequency) of each population analyzed (+/- SEM) and the gate used for analysis.
